# The Middle Classes in Hospital

**Published:** 1923-05

**Authors:** 


### THE MIDDLE CLASSES IN HOSPITAL.

SIR NAPIER BURNETT announced recently that he is working out a scheme of insurance which would enable the middle and upper classes to enjoy the " inestimable benefits " of hospital treatment. Such a scheme, he believes, would be of great advantage both to the public and the hospitals and would, at the same time, provide means for the remuneration of the medical men. The details of the plan will be awaited with peculiar interest by all who realise the growing importance of the hospitals, not merely as institutions for the cure of disease, but as wonderful centres of discovery for the prevention of disease. Prevention is the great work of the future, and the more money and the more experience the hospitals acquire the more rapidly and certainly will that work progress. In the meantime there is everything to be said for extending the benefits of hospital treatment to classes other than those which at present chiefly take advantage of them.

The old idea that a hospital is a charity pure and simple is exploded. For the destitute sick it will ever be an asylum and the tribute which the more fortunate pay to poverty and weakness. For works of mercy there will always be room, but works of mercy need not necessarily be confined to the succour of the afflicted who are both poor and ill. The relatively well-to-do are often in very unhappy case when serious illness, especially illness involving surgical aid, overtakes them. They are virtually compelled to go into nursing homes, which are nearly always costly and by no means always satisfactory. They are good and they are bad; still more often they are indifferent. The best are splendid; the worst abominable, and we have little right to tolerate the indifferent. Those which are under the control of medical men of first-rate attainments are in an entirely different position from those in which specialist supervision is more or less lacking. In the hospitals that supervision is everywhere and is never relaxed. But now that we are asking the respectable working man to take out an insurance entitling him and his family to the ever-increasing advantages of hospital treatment, we are surely entitled to suggest that other classes should, in their own interest, follow suit. The professional man, for instance, is relatively as hard hit by grievous illness as the manual worker, and it is only by heavy expenditure that he can secure the best treatment and the best surgery.

The advantages of insurance of this kind are as obvious in the one case as in the other. If a practicable plan for securing hospital treatment for those for whom it has hitherto as a rule been unavailable can be devised, a highly important work will have been accomplished for the nation as well as for the hospitals. It may not be easy to devise it, and success must in great measure depend upon the extent to which advantage is taken of it, but success would produce a revolution of a much more beneficent character than most revolutions. And, in so far as hospitals are concerned, we are living in highly revolutionary times.

